# The synergetic effect of sitafloxacin–arbekacin combination in the *Mycobacterium abscessus* species

**DOI:** 10.1038/s41598-023-29021-0

**Published:** 2023-02-04

**Authors:** Junko Watanabe, Hiroaki Ihara, Satomi Takei, Ayako Nakamura, Yuichi Fujimoto, Tetsuya Handoh, Kana Kurokawa, Yuta Arai, Kohei Shibayama, Issei Sumiyoshi, Yusuke Ochi, Takahiro Okabe, Shigeki Misawa, Shinsaku Togo, Toshio Naito, Yoko Tabe, Takashi Miida, Kazuhisa Takahashi

**Affiliations:** 1 Department of Respiratory Medicine, Graduate School of Medicine, Juntendo University, Faculty of Medicine, 2-1-1 Hongo, Bunkyo-ku, Tokyo, 113-8421 Japan; 2 Department of Clinical Laboratory Medicine, Graduate School of Medicine, Juntendo University, Faculty of Medicine, Tokyo, Japan; 3 Research Institute for Diseases of Old Ages, Graduate School of Medicine, Juntendo University, Faculty of Medicine, Tokyo, Japan; 4 Leading Center for the Development and Research of Cancer Medicine, Graduate School of Medicine, Juntendo University, Faculty of Medicine, Tokyo, Japan; 5 Department of General Medicine, Graduate School of Medicine, Juntendo University, Faculty of Medicine, Tokyo, Japan; 6 Koto Hospital, Tokyo, Japan; 7 Juntendo Tokyo Koto Geriatric Medical Center, Tokyo, Japan

**Keywords:** Microbiology, Antimicrobials

## Abstract

*Mycobacterium abscessus* species (MABS) is the most commonly isolated rapidly growing mycobacteria (RGM) and is one of the most antibiotic-resistant RGM with rapid progression, therefore, treatment of MABS is still challenging. We here presented a new combination treatment with sitafloxacin that targeted rough morphotypes of MABS*,* causing aggressive infections. Thirty-four clinical strains of MABS were isolated from various clinical samples at the Juntendo university hospital from 2011 to 2020. The susceptibility to a combination of sitafloxacin and antimicrobial agents was compared to that of the antimicrobial agents alone. Out of 34 MABS, 8 strains treated with sitafloxacin–amikacin combination, 9 of sitafloxacin–imipenem combination, 19 of sitafloxacin–arbekacin combination, and 9 of sitafloxacin–clarithromycin combination showed synergistic effects, respectively. Sitafloxacin–arbekacin combination also exhibited the synergistic effects against 10 of 22 *Mycobacterium abscessus* subspecies *massiliense* (Mma) strains and 8 of 11 *Mycobacterium abscessus* subspecies *abscessus* (Mab) strains*,* a highly resistant subspecies of MABS. The sitafloxacin–arbekacin combination revealed more synergistic effects in rough morphotypes of MABS (*p* = 0.008). We demonstrated the synergistic effect of the sitafloxacin–arbekacin combination against MABS. Further, this combination regimen might be more effective against Mab or rough morphotypes of MABS.

## Introduction

Nontuberculous mycobacteria (NTM) are environmental pathogens that can cause diverse types of infectious diseases in humans. NTM are classified into RGM where colony formation requires less than seven days and slowly growing mycobacteria (SGM) forming colonies at least seven days. MABS is the most commonly isolated RGM and the third most common cause of respiratory NTM in the United States^1^. Pulmonary disease caused by MABS mostly occur in the setting of structural lung conditions. Most of the patients underlying disease in Japan were bronchiectasis, chronic obstructive pulmonary disease (COPD), previous pulmonary tuberculosis; whereas, that in North America and Europe was cystic fibrosis (CF)^2–4^. These infections are often incurable and associated with rapid lung function decline^5,6^. New NTM treatment guidelines were published in 2020^7^. The guidelines introduced new treatment options, including inhaled amikacin, tigecycline, and clofazimine^8–10^; however, the treatment benefits were limited to negative culture conversion of sputum. Recently, the efficacy of sitafloxacin, a fluoroquinolone developed in Japan, containing regimens against MABS have been reported^11,12^. Sitafloxacin, with a chloro substituent at the C-8 position, is a newly developed oral quinoline, exhibiting good antimicrobial activity against extracellular and intramacrophage *Mycobacterium avium* complex (MAC) compared to levofloxacin in vitro and in vivo^13–16^. These previous papers suggest that fluoroquinolone combining regimens could have a potency for the effective treatment of MABS. Genus mycobacterium included *Mycobacterium tuberculosis* (*M. tuberculosis*), and some NTM have long been known to have both rough and smooth colony morphotypes^17,18^. These morphotypes are formed by the expression levels of glycopeptidolipids (GPLs). GPLs are produced by several NTMs, including RGMs (*M. abscessus*, *M. chelonae,* and *M. smegmatis*)^19–21^ and MAC members^22–24^. MABS can spontaneously change between a smooth form, which expresses GPLs, and a rough form, lacking GPLs. The smooth form can form biofilms and colonize surfaces; conversely, the rough morphotypes cannot form biofilms but can multiply in macrophages and cause persistent infection^25^. Rough morphotypes are generally more virulent than smooth variants for isolates lacking GPLs enhanced releasing TNF-α from macrophage^25–27^. Conversely, to form biofilms, smooth variants were related to protecting from surrounding factors^28^. Here, we presented the new sitafloxacin–arbekacin combination regimens, which are more effective on rough morphotypes, causing aggressive infections, and could be a potential treatment option against Mab.

## Results

**Figure 1:**
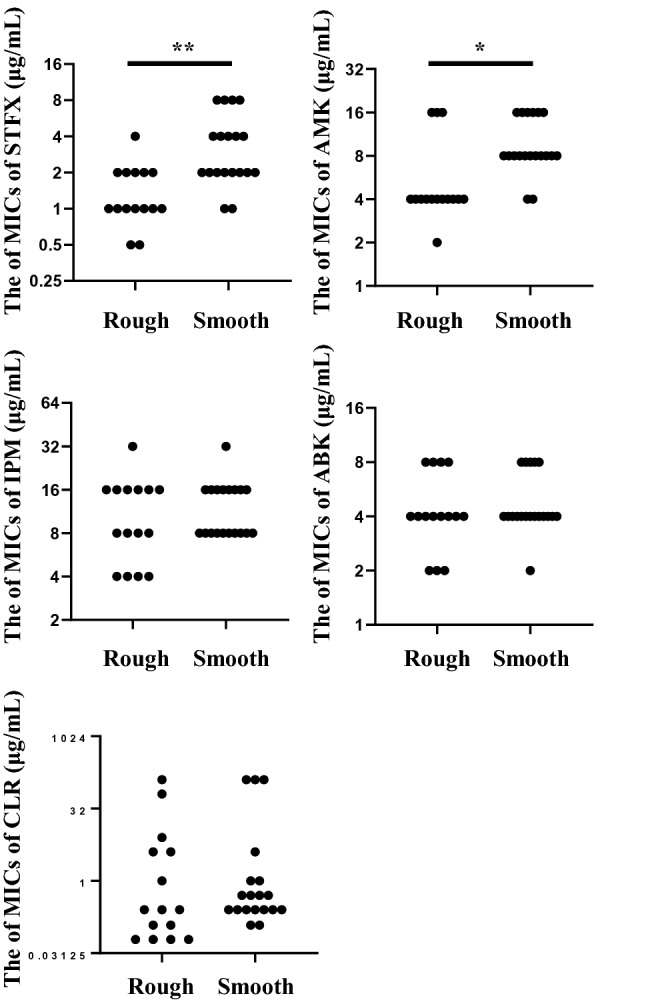
The comparison of MIC of each antimicrobial, STFX, intravenous AMK, IPM, ABK, and CLR, compared with rough and smooth colony morphotypes. *p value < 0.05, **p value < 0.01. *STFX* sitafloxacin, *AMK* amikacin, *IPM* imipenem, *ABK* arbekacin, *CLR* clarithromycin.

**Figure 2:**
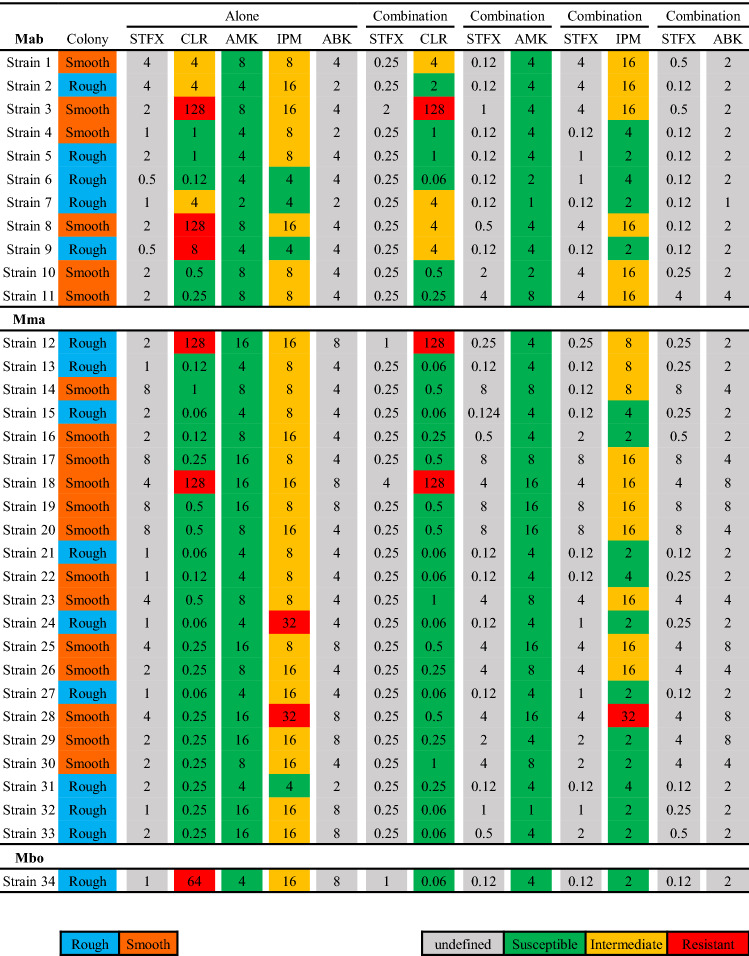
MIC distributions for intravenous AMK, IPM, and ABK combined with STFX, categorized into three subspecies of MABS on day 7. Light blue color indicates rough colony morphotype, and orange color smooth colony morphotype. Green color indicates susceptibility, yellow color intermediate, and red color resistance to MABS. Gray color indicates MIC breakpoints undefined. *STFX* sitafloxacin, *AMK* amikacin, *IPM* imipenem, *ABK* arbekacin, *CLR* clarithromycin, *Mma* *Mycobacterium abscessus* subspecies *massiliense*, *Mab* *Mycobacterium abscessus* subspecies *abscessus*, *Mbo* *Mycobacterium abscessus* subspecies *bolletii*, *MABS* *Mycobacterium abscessus* species.

**Figure 3:**
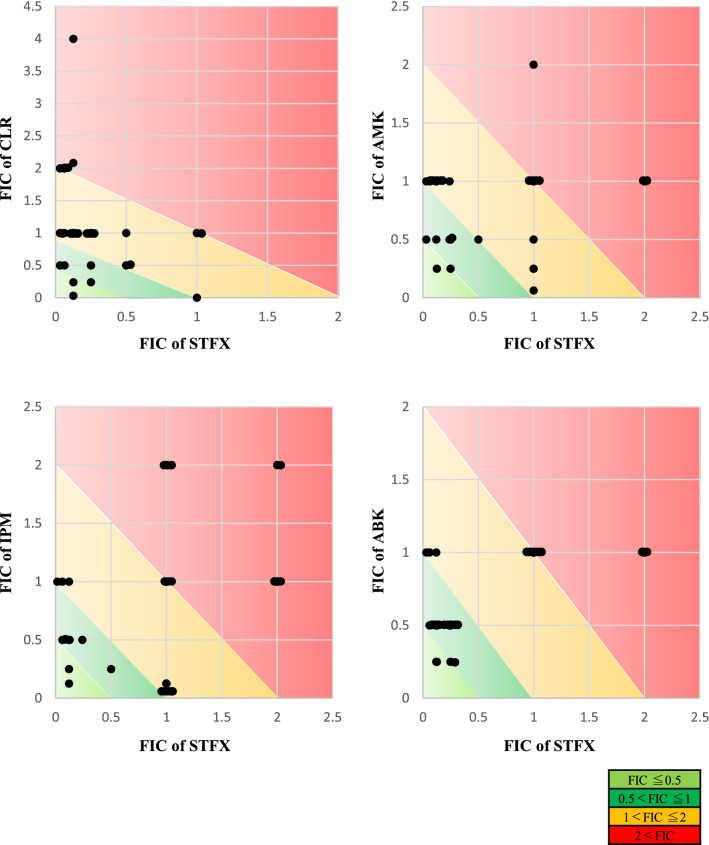
The relation between FIC of sitafloxacin and that of the other antibiotics. Light green color indicates ≤ 0.5 of FIC value, green color indicates 0.5 < to 1, yellow color indicates 1 < to 2, and red color indicates 2< . *STFX* sitafloxacin, *AMK* amikacin, *IPM* imipenem, *ABK* arbekacin, *CLR* clarithromycin, *FIC* fractional inhibitory concentration.

**Figure 4:**
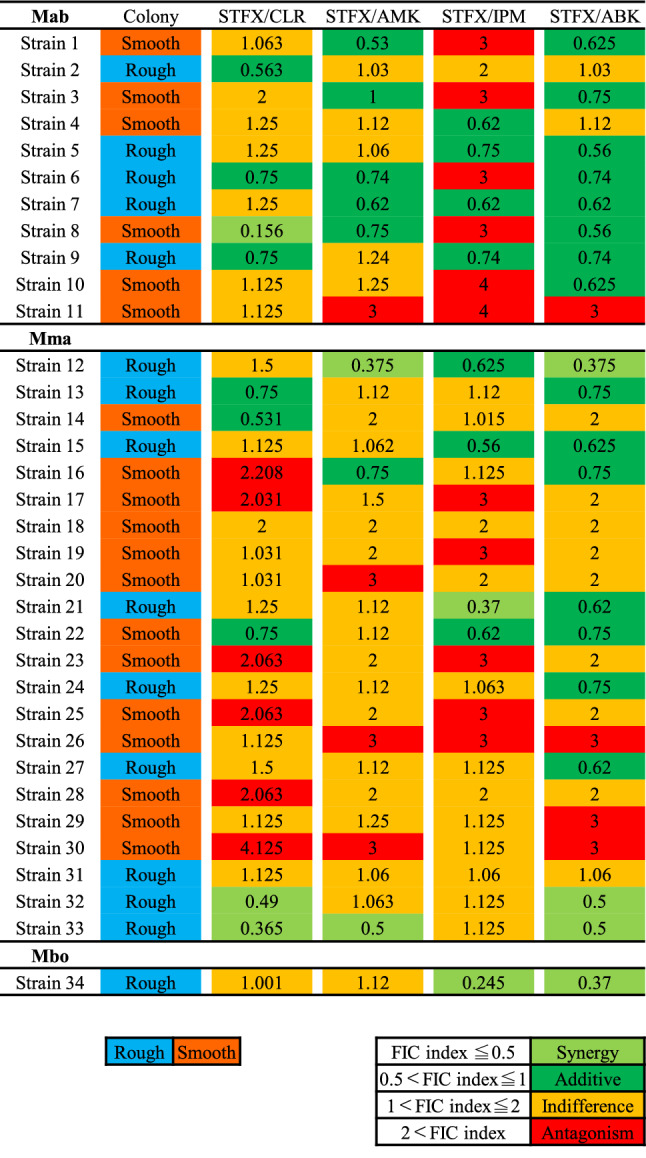
FIC index of intravenous AMK, IPM, ABK, and CLR combined with STFX categorized into three subspecies of MABS. Light blue color indicates rough colony morphotype, and orange color smooth colony morphotype. Light green color indicates synergy, green color indicates additive, yellow color indicates indifference, and red color indicates antagonism in each combination. *STFX* sitafloxacin, *AMK* amikacin, *IPM* imipenem, *ABK* arbekacin, *CLR* clarithromycin, *FIC index* fractional inhibitory concentration index, *Mma* *Mycobacterium abscessus* subspecies *massiliense*, *Mab* *Mycobacterium abscessus* subspecies *abscessus*, *Mbo* *Mycobacterium abscessus* subspecies *bolletii*, *MABS* *Mycobacterium abscessus* species.

Thirty-four clinical strains of MABS were isolated from various clinical samples at the Juntendo university hospital from 2011 to 2020. The characteristics of patients isolated from MABS are shown in Table 1. Methods of incubation time and susceptibility testing were used as a reference to our previous report^29^. Five antimicrobials (clarithromycin, intravenous amikacin, imipenem, arbekacin, and sitafloxacin) were used for the study. The difference of MICs between both colony morphotypes was evaluated (Fig. 1), and MICs of sitafloxacin and intravenous amikacin in rough morphotypes were significantly lower than smooth morphotypes (*p* values of sitafloxacin and intravenous amikacin were 0.0004 and 0.002, respectively). Therefore, we investigated the best combination partners of sitafloxacin as the potential regimens for MABS especially rough morphotypes. The susceptibility to a combination of sitafloxacin and antimicrobial agents was compared to that of the antimicrobial agents alone, categorized into each subspecies of MABS (Fig. 2). The MICs of four antimicrobial agents (clarithromycin, intravenous amikacin, imipenem, and arbekacin) were measured with or without sitafloxacin. Ten of 11 Mab were susceptible to sitafloxacin in the combination administration*;* while, 11 of 22 Mma were susceptible. The median MICs of sitafloxacin and arbekacin in MABS were significantly lower in the combination administration (*p* values of sitafloxacin and intravenous amikacin were < 0.001 and 0.028, respectively, Table S2). We next evaluated the most synergistic combinations by using the fractional inhibitory concentration (FIC) index as described in previous paper^30^. Figure 3 showed the relation between FIC of sitafloxacin and that of the other antibiotics. The combination of sitafloxacin and amikacin tended to be obviously higher rate of synergy and additive effect. Further evaluation of FIC index of each combination was performed (Fig. 4 and Table 2). Susceptibility was divided into two classes, synergy and additive as a synergistic effect and indifference and antagonism as an antagonistic effect. Out of 34 MABS, 8 strains treated with sitafloxacin–amikacin combination, 9 of sitafloxacin–imipenem combination, 19 of sitafloxacin–arbekacin combination, and 9 of sitafloxacin–clarithromycin combination showed synergistic effects, respectively. Sitafloxacin–arbekacin combination also exhibited the synergistic effects against 10 of 22 Mma strains and 8 of 11 Mab strains*,* a highly resistant subspecies of MABS*.* We investigated whether susceptibility to the sitafloxacin–arbekacin combination might associate with clinical or isolate status. The rough colony morphotypes revealed more synergistic effects than antagonistic effects (*p* = 0.008) (Table 3). The other clinical parameters such as age, sex, smoking history, bronchiectasis lesion, and treatment history of antibiotics did not influence the sitafloxacin–arbekacin combination.

**Table 1:** The characteristics of patients from which MABS were isolated.

	N = 34
Sex (male/female)	16/18
Median age (range)	65.5 (30–83)
Smoking history, N (%)	13 (38.2)
MABS subtype, N (%)	
*Mycobacterium abscessus* subsp. *abscessus*	11 (32.4)
*Mycobacterium abscessus* subsp. *masiliense*	22 (64.7)
*Mycobacterium abscessus* subsp. *Bolletii*	1 (2.9)
Colony phenotype (rough/smooth)	15/19
MABS detected from, N (%)	
Sputum or bronchial lavage	27 (79.4)
Others	7 (20.6)
Pretreatment of antibiotics within 3 months, N (%)	
Macrolides	5 (14.7)
Fluoroquinolones	4 (11.8)
Tetracyclines	2 (5.9)
Others	12 (35.3)
Comorbidity, N (%)	
Bronchiectasis	14 (41.2)
Diabetes mellitus	4 (11.8)
Immunodeficiency (non HIV)	2 (5.9)
Malignancy	7 (20.6)
Concomitant medications, N (%)	
Corticosteroids	6 (17.6)
Immunosupressant	3 (8.8)

*HIV* human immunodeficiency virus, *MABS* *Mycobacterium abscessus* species.

**Table 2:** The number of synergistic and antagonistic combination with STFX and each antimicrobial.

Species	Categories of FIC index		STFX/CLR		STFX/AMK		STFX/IPM		STFX/ABK		*p* value
			N (%, adjusted residual)								
MABS	FIC index ≤1		9 (26.5, −0.9)		8 (23.5, −0.9)		9 (26.5, −1.4)		19 (55.9, 3.3**)		0.016*
N = 34^a^	FIC index 1 <		25 (73.5, 0.9)		26 (76.5, 0.9)		25 (73.5, 1.4)		15 (44.1, −3.3**)		
Mma	FIC index ≤1		5 (22.7, −0.3)		3 (13.6, −1.4)		4 (18.2, −0.9)		10 (45.5, 2.6)		0.083
N = 22	FIC index 1 <		17 (77.2, 0.3)		19 (86.4, 1.4)		18 (81.8, 0.9)		12 (54.5, −2.6)		
Mab	FIC index ≤1		4 (36.4, −0.9)		5 (45.5, −0.2)		4 (36.4, −0.9)		8 (72.7, 1.9)		0.26
N = 11	FIC index 1 <		7 (63.6, 0.9)		6 (54.5, 0.2)		7 (63.6, 0.9)		3 (27.3, −1.9)		

*FIC index* fractional inhibitory concentration index, *STFX* sitafloxacin, *CLR* clarithromycin, *AMK* amikacin, *IPM* imipenem, *ABK* arbekacin, *MABS* *Mycobacterium abscessus* species, Mma, *Mycobacterium abscessus* subspecies *massiliense*; Mab, *Mycobacterium abscessus* subspecies *abscessus*; Mbo, *Mycobacterium abscessus* subsp.*boletii.*

*p value < 0.05, **p value < 0.01.

*Adjusted residuals >|1.96|, **adjusted residuals >|2.58|.

^a^Including Mbo(n = 1).

**Table 3:** The number of synergistic and antagonistic combination with STFX and ABK in each clinical status.

	FIC index		
	Synergy + additive, N = 19 (%)	Indifference + antagonism, N = 15 (%)	*p* value
Age			
< 65 years	7 (20.6)	7 (20.6)	0.56
≥ 65 years	12 (35.3)	8 (23.5)	
Sex			
Male	11 (32.4)	5 (14.7)	0.15
Female	8 (23.5)	10 (29.4)	
Smoking history			
Yes	7 (20.6)	6 (17.6)	0.85
No	12 (35.3)	9 (26.5)	
With bronchiectasis			
Yes	8 (23.5)	6 (17.6)	0.90
No	11 (32.4)	9 (26.5)	
With immunosuppression			
Yes	8 (23.5)	8 (23.5)	0.51
No	11 (32.4)	7 (20.6)	
Pretreatment of antibiotics			
Yes	8 (23.5)	6 (17.6)	0.90
No	11 (32.4)	9 (26.5)	
Colony morphotypes			
Smooth	6 (17.6)	13 (38.2)	0.008**
Rough	13 (38.2)	2 (5.9)	

Antibiotics including CLR (n = 3).

*FIC index* fractional inhibitory concentration index, *STFX* sitafloxacin, *ABK* arbekacin, *CLR* clarithromycin.

*p value < 0.05, **p value < 0.01.

## Discussion

We demonstrated here the efficacy of the new sitafloxacin–arbekacin combination regimen. The combination addministration revealed MIC reduction of sitafloxacin and arbekacin, and significantly high synergistic effect against MABS. The combination regimen showed a higher rate of susceptibility and synergistic effects against Mab than all other combinations. Interestingly, the combination had relatively higher efficacy in Mma than others including clarithromycin–arbekacin combination, even though clarithromycin is the key drug of Mma treatment. These results might suggest that the combination therapy was more effective against Mab, showing a high level of antimicrobial resistance. Furthermore, the combination revealed higher efficacy for the treatment of MABS in rough morphotypes associated with aggressive infections.

Sitafloxacin is approved in Japan, and it has been clinically used against most NTM infections. Formerly, sitafloxacin has been mainly used for MAC infections among NTMs; then, in vitro studies and clinical use for MABS has increased. Bedaquiline-clofazimine-sitafloxacin combination revealed a synergistic effect against 11 isolates of 70 Mab (15.7%)^11^. In a Japanese retrospective study of 13 MABS pulmonary disease, all 4 patients who received sitafloxacin-containing regimens achieved negative sputum conversion after 1 year of treatment and improved radiological findings^12^. Japanese case series described that five cases of pulmonary MABS were successfully treated with clarithromycin and sitafloxacin combination^31^. Together with our data and previous reports, sitafloxacin could be an effective antimicrobial combination partner against refractory MABS. MABS exist in two distinct morphotypes, smooth and rough, that differ in their gross colony appearances when grown on solid media due to their differing amounts of cell wall GPLs. The smooth morphotype initially colonizes the airway mucosa, and had generally lower pathogenicity in this state. Subsequently switching from smooth morphotypes to rough morphotypes, aggressive pulmonary disease cause. Smooth morphotypes have an advantage in survival due to biofilm formation, leading to inhibit bacteria-induced apoptosis^32^. Conversely, rough morphotypes, without biofilms, induce the invasion ability mediated by apoptotic cell death^33,34^. Several clinical data have revealed increased pathogenicity from the rough morphotype. The rates of isolation of rough morphotype is higher in the CF patients with clinical symptoms^35^, and case reports describe that the CF patients with rough morphotypes lead to dramatic declines of respiratory function and/or death^26,36^. Thus, the development of new treatment targeted rough morphotypes has become imperative. Our study revealed that sitafloxacin–arbekacin combination had the higher synergy in rough morphotypes, the combination could be useful treatment for the patients who are isolated with rough morphotypes and/or whose disease have progressed. Interestingly, in 17 out of 19 smooth morphotypes, sitafloxacin susceptibility in the combination treatment improved as compared to alone. This data suggested that sitafloxacin–arbekacin combination could be also partially effective as the treatment for smooth morphotypes of MABS. In conclusion, our in vitro study demonstrated the synergistic effect of the sitafloxacin–arbekacin combination against MABS. Further, this combination regimen might be more effective against not only rough morphotype of MABS, causing severe disease, but also Mab, which is thought to reveal high resistance to antibiotics. The limitation of our study is that the sample size was limited to make definitive concerns and the clinical efficacy of the sitafloxacin–arbekacin combination have not been assessed. Further studies are required to clarify the validity of the combination.

## Materials and methods

### Determination of MABS

Three subspecies of MABS was confirmed by sequencing the *16S rRNA*, *rpoB*, *hsp65*, and *erm* genes^37,38^. All strains of MABS were cultured on BD trypticase soy agar II with 5% sheep blood (Blood agar; Nippon Becton–Dickinson and Company, Japan) at 35 °C for approximately 4 to 6 days to observe colony morphology and purity and then used for species identification based on multi-locus sequence analysis. Colony morphologies were confirmed before the drug sensitivity testing, and all strains grew to maintain the same colony morphologies even after repeat-passage.

Methodological details are described in the Supplementary Materials and Methods.

### PCR amplification, DNA sequencing, and MALDI–TOF MS analysis

The conditions of each analysis and primer sequences used in PCR to detect transcripts are described in the Supplementary materials and methods.

### Antimicrobial susceptibility testing

Susceptibility testing was performed according to Clinical and Laboratory Standard Institute (CLSI) guideline M24-A2^35^. The bacterial suspension was diluted at a concentration of 1–5 × 10^5^ colony forming units (CFU)/mL in cation-adjusted Mueller–Hinton broth (CAMHB), then the final suspension was inoculated on the break-point checkerboard plate customized for the study (Eiken Chemical Co., Ltd., Japan). The ranges of antibiotic concentrations tested were as follows: clarithromycin (CLR) 0.06 to 64 μg/mL, arbekacin (ABK) 1 to 8 μg/mL, intravenous amikacin (AMK) 1 to 64 μg/mL, imipenem (IPM) 2 to 32 μg/mL, and sitafloxacin (STFX) 0.12 to 32 μg/mL. MICs of each antimicrobial agent were determined by broth microdilution methods as recommended by the CLSI. The panels were prepared with a 96-channel dispenser and stored at −80 °C until use. Sitafloxacin were dispensed alone in the first row, and arbekacin, intravenous amikacin, imipenem were dispensed in the first column. Each well was inoculated with a concentration of 1 × 10^5^ colony-forming units (CFU)/mL. The MICs were determined after 7 days of incubation at 35 °C. The MIC breakpoints, indicating susceptible, intermediate, and resistant strains, were interpreted according to the Clinical and Laboratory Standard Institute (CLSI) criteria (Table 4)^39^. Sitafloxasin and arbekacin breakpoints were undefined. The effect of each agent combined with sitafloxacin was evaluated using FIC index analysis^30^.

**Table 4:** Antimicrobial agents and MIC breakpoints for RGM.

Antimicrobial agents	MIC (μg/mL) for category		
	Susceptible	Intermediate	Resistant
Amikacin	≤ 16	32	≥ 64
Cefoxitin	≤ 16	32–64	≥ 128
Ciprofloxacin	≤ 1	2	≥ 4
Clarithromycin	≤ 2	4	≥ 8
Doxycycline	≤ 1	2–4	≥ 8
Imipenem	≤ 4	8–16	≥ 32
Linezolid	≤ 8	16	≥ 32
Moxifloxacin†	≤ 1	2	≥ 4
Trimethoprim-sulfamethoxazole	≤ 2/38	–	≥ 4/76
Tobramycin	≤ 2	4	≥ 8

Sitafloxacinand arbekacin breakpoints were undefined.

*RGM* rapidly growing mycobacteria.

### Statistical analysis

Categorical variables were compared using the chi-square test or Fisher's exact test. The evaluation of changes in MIC was performed using the Wilcoxon signed-rank test. Differences were considered significant at *p* < 0.05. When the chi-square test results were statistically significant, adjusted residuals were calculated to determine which particular associations were significant. Adjusted residuals were significant at *p* < 0.05 level if they were less than − 1.96 or more than 1.96 and were significant at *p* < 0.01 level if they were less than − 2.58 or more than 2.58. All statistical analyses were performed using the SPSS software program (version 20, IBM Japan, Japan).

## Supplementary Information

Supplementary Information.

## Data Availability

The datasets used in the current study are available from the corresponding author on reasonable request.

## Abbreviations

MABS: *Mycobacterium **abscessus* Species
NTM: Nontuberculous mycobacteria
RGM: Rapidly growing mycobacteria
SGM: Slowly growing mycobacteria
Mma: *Mycobacterium **abscessus* Subspecies *massiliense*
Mab: *Mycobacterium **abscessus* Subspecies *abscessus*
Mbo: *Mycobacterium **abscessus* Subspecies *bolletii*
*M. tuberculosis*: *Mycobacterium tuberculosis*
COPD: Chronic obstructive pulmonary disease
CF: Cystic fibrosis
GPL: Glycopeptidolipid
MAC: *Mycobacterium avium* Complex
MALDI-TOF MS: Matrix-assisted laser-desorption/ionization time-of-flight mass spectrometry
CLSI: Clinical and Laboratory Standard Institute
STFX: Sitafloxacin
AMK: Amikacin
IPM: Imipenem
ABK: Arbekacin
HIV: Human immunodeficiency virus
FIC: Fractional inhibitory concentration
MBEC: Minimum biofilm eradication concentration
CAMHB: Cation-adjusted Mueller–Hinton broth
CFU: Colony forming units
