# Supplementary material for: The synergetic effect of sitafloxacin–arbekacin combination in the Mycobacterium abscessus species
Source: Sci Rep. 2023 Feb 4;13:2027. doi: 10.1038/s41598-023-29021-0 (PMC9899205; doi:10.1038/s41598-023-29021-0)
Supplement: Supplementary file 1 — Supplementary Information. [file 41598_2023_29021_MOESM1_ESM.pdf]

Supplementary Material and Methods

Determination of MAB*S*

All material samples suspected of mycobacterial contamination in the Juntendo university hospital were cultured in a mycobacteria growth indicator tube (MGIT; Becton Dickinson, USA) broth and incubated at 37°C in the BACTEC MGIT 960 (Becton Dickinson, USA) instrument with ambient air. MGIT positive tubes were classified as *M. abscessus* based on the results of DNA–DNA hybridization (DDH) analysis (DDH Mycobacterium "Kyokuto" kit; Kyokuto Pharmaceutical Industrial, Japan) or matrix-assisted laser-desorption/ionization time-of-flight mass spectrometry (MALDI-TOF MS).

PCR amplification, DNA sequencing, and MALDI-TOF MS analysis

DNA was extracted from cultured colonies using the DNeasy UltraClean Microbial Kit (QIAGEN, Germany), and PCR was conducted using Ex Taq DNA polymerase, hot-start version (Takara, Japan) according to the manufacturer's instructions. The gene-specific primer pairs used for PCR analysis are listed in table S1; these primers were used in previous studies [1,2]. The sequencing PCR products were purified with the BigDye XTerminator purification kit (Life Technologies, USA), and samples were loaded

on the ABI Prism 3130 Genetic Analyzer (Thermo Fisher Scientific, USA). The DNA sequencing results were analyzed using a BLAST search to identify sequence similarity between samples and the three species of MABS.

MALDI-TOF MS analysis was performed based on previously described methods [3]. Colonies of MABS on blood agar were scratched with a needle, and particles on the needle surface were diluted in 50 μL 80% trifluoroacetic acid. After incubation for 15 minutes at room temperature, the solution was added to 150 μL distilled water and 200 μL 100% acetonitrile, followed by a centrifugation step (16,200 × g, 2 min). One microliter of the cleared supernatant containing the bacterial extract was transferred onto a MALDI target plate (Bruker Daltonik, Germany). We overlaid dried spots with MALDI matrix (10 mg/mL α-cyano-4-hydroxy cinnamic acid [α-HCCA] in 50% acetonitrile:2.5% trifluoroacetic acid) (Bruker Daltonik, Germany). After drying the matrix, we conducted MALDI-TOF MS analysis with a Microflex LT/SH benchtop mass spectrometer (Bruker Daltonik, Germany) equipped with a 60-Hz nitrogen laser. We had optimized parameter settings (ion source 1 [IS1], 20 kV; IS2, 18.2 kV; lens, 6.85 kV; detector gain, 2854 V; gating, none) for the mass range between 2,000 and 20,000 Da. We achieved spectra in the positive linear mode with the maximum laser frequency. An external standard (bacterial test standard [BTS]) (Bruker Daltonik, Germany) was applied for instrument calibration. Data

evaluation was performed by visually comparing spectra to search for peak shifts using flexAnalysis 3.4 (Bruker Daltonik, Germany).

Reference

1. Nakanaga K, Sekizuka T, Fukano H, et al. Discrimination of Mycobacterium abscessus subsp. massiliense from Mycobacterium abscessus subsp. abscessus in clinical isolates by multiplex PCR. *J Clin Microbiol.* 2014;52(1):251-259.
2. Brown-Elliott BA, Vasireddy S, Vasireddy R, et al. Utility of sequencing the erm(41) gene in isolates of Mycobacterium abscessus subsp. abscessus with low and intermediate clarithromycin MICs. *J Clin Microbiol.* 2015;53(4):1211-1215.
3. Sparbier K, Lange C, Jung J, Wieser A, Schubert S, Kostrzewa M. MALDI biotyper-based rapid resistance detection by stable-isotope labeling. *J Clin Microbiol.* 2013;51(11):3741-3748.

Supplementary Table

Supplementary Table 1: Forward and backward primers used for PCR

**Target**	**Sequence**
16S rRNA	Forward, 5’-AGA GTT TGA TCM TGG CTC AG-3’
	Reverse, 5’-TAC GGT TAC CTT GTT ACG AC-3’
rpoB	Forward, 5’-GAG GGT CAG ACC ACG ATG AC -3’
	Reverse, 5’-AGC CGA TCA GAC CGA TGT T-3’
hsp65	Forward, 5’ACC AAC GAT GGT GTG TCC AT -3’
	Reverse, 5’ CTT GTC GAA CCG CAT ACC CT-3’
erm	Forward, 5’-GAC CGG GCC TTC GTG AT -3’
	Reverse, 5’-GAC TTC CCC GCA CCG ATT CC-3’

Supplementary Table 2: The changes of median MIC of STFX and ABK between monotherapy and combination therapy.

Species	Median MIC of STFX (monotherapy)	Median MIC of STFX (combination therapy)	*p* value	Median MIC of ABK (monotherapy)	Median MIC of ABK (combination therapy)	*p* value
MABS N=34†	2	0.25	0.028*	4	2	<0.001**
Mma N=22	2	2.25	0.42	4	3	0.002**
Mab N=11	2	0.12	0.031*	4	2	0.008*

†Including Mbo (n=1)

**p* value <0.05, ** *p* value <0.01

Abbreviations: STFX, sitafloxacin; ABK, arbekacin; Mbo, *Mycobacterium abscessus* subspecies *boletii*
